# Daily rhythms in plasma levels of homocysteine

**DOI:** 10.1186/1740-3391-2-5

**Published:** 2004-09-03

**Authors:** Lena Lavie, Peretz Lavie

**Affiliations:** 1 Unit of Anatomy and Cell Biology, Bruce Rappaport Faculty of Medicine, Technion-Israel Institute of Technology, Haifa 31096, Israel

## Abstract

**Background:**

There is accumulated evidence that plasma concentration of the sulfur-containing amino-acid homocysteine (Hcy) is a prognostic marker for cardiovascular morbidity and mortality. Both fasting levels of Hcy and post methionine loading levels are used as prognostic markers. The aim of the present study was to investigate the existence of a daily rhythm in plasma Hcy under strictly controlled nutritional and sleep-wake conditions. We also investigated if the time during which methionine loading is performed, i.e., morning or evening, had a different effect on the resultant plasma Hcy concentration.

**Methods:**

Six healthy men aged 23–26 years participated in 4 experiments. In the first and second experiments, the daily rhythm in Hcy as well as in other amino acids was investigated under a normal or an inverse sleep-wake cycle. In the third and fourth, Hcy concentrations were investigated after a morning and evening methionine loading. To standardize food consumption in the first two experiments, subjects received every 3 hours 150 ml of specially designed low-protein liquid food (Ensure^® ^formula).

**Results:**

In both the first and second experiments there was a significant daily rhythm in Hcy concentrations with a mid-day nadir and a nocturnal peak. Strikingly different 24-h patterns were observed in methionine, leucine, isoleucine and tyrosine. In all, the 24-h curves revealed a strong influence of both the sleep-wake cycle and the feeding schedule. Methionine loading resulted in increased plasma Hcy levels during both morning and evening experiments, which were not significantly different from each other.

**Conclusions:**

There is a daily rhythm in plasma concentration of the amino acid Hcy, and this rhythm is independent of sleep-wake and food consumption. In view of the fact that increased Hcy concentrations may be associated with increased cardiovascular risks, these findings may have clinical implications for the health of rotating shift workers.

## Background

Experimental results accumulated in recent years have revealed that plasma concentration of the sulfur-containing amino-acid homocysteine (Hcy) is a prognostic marker for cardiovascular morbidity and mortality [1-5]. Plasma concentrations of Hcy in excess of 15 μmol/L under fasting conditions were associated with increased risk of cardiovascular mortality [6]. Furthermore, some patients having normal fasting levels of plasma Hcy were shown to have abnormally high levels of Hcy after methionine loading [7]. In most epidemiological studies, the differences between fasting concentrations of Hcy of cardiovascular patients and normal controls did not amount to more than 10–15%.

Studies conducted during the 1960s have demonstrated that plasma levels of several amino acids vary in a daily manner. Feigin, Klainer and Beisel [8] were the first to report on daily rhythms in serum levels of total amino acids in adult men. The peak levels of the total integrated amino acids occured between 1200 and 2000 with a minimum level at 0400. Wurtman, Chou and Rose [9] reported on a daily rhythm in plasma concentration of tyrosine with a nocturnal nadir and a morning peak, which represented a two-fold increase in plasma tyrosine level. This rhythm persisted when subjects were maintained on a two-week low protein diet. Subsequently, the same group [10] extended their findings to 15 additional amino acids. Tyrosine, tryptophan, phenylalanine, methionine, cysteine, and isoleucine, underwent the greatest daily changes while alanine, glycine and glutamic acid showed the least. Hussein et al [11] reported that the daily fluctuations of plasma free amino acids were significantly affected by the dietary conditions. In none of these studies, however, were the levels of amino acids determined during the sleep period or under uniform dietary conditions.

More recently, plasma Hcy levels were also shown to vary in a daily manner in humans with an evening peak and a morning nadir [12]. Significant daily rhythmicity was found in obese diabetic patients but not in normal controls. Since plasma samples were obtained every 3 hours and no attempt was made to examine how sleep affected the pattern of secretion, it is difficult to determine whether these findings bear any clinical significance. In rats, plasma Hcy demonstrated a 24-h rhythm with a nocturnal peak and a daytime nadir. Pinealectomy did not change the phase of the rhythm or its nocturnal elevation, but it did significantly increase mean plasma Hcy [13].

In the present study, we further investigated the possible existence of a daily rhythm in plasma Hcy under strictly controlled nutritional and sleep-wake conditions. We also investigated if the time during which methionine loading is performed, i.e., morning or evening, had a different effect on the resultant plasma Hcy concentration.

## Methods

### Subjects

Six healthy men aged 23–26 years participated in 4 experiments. All were students who maintained a normal and regular sleep-wake cycle for at least three months prior to the studies. They were screened to ensure an adequate state of health by physical examination, detailed medical history and blood testing. All had a normal body weight (mean body mass index (BMI) = 23.5 ± 1.6 Kg/m^2^). They were instructed to avoid alcohol and coffee beverages during the 24 hours that preceded each of the experimental periods. The study was approved by the local Human Ethics Committee, and subjects gave written informed conset before being enrolled in the first experiment. Subjects were paid for their participation.

### Procedure

In the first and second experiments, daily rhythms in Hcy as well as in other amino acids were investigated under a normal or an inverse sleep-wake cycle. In the third and fourth experiments, Hcy concentrations were investigated after morning and evening methionine loading.

### Experiment 1

Subjcts were admitted to the laboratory at 1800 for a period of 24 hours, after having a normal day. A catheter was inserted into an antecubital vein and was kept patent by a drip of saline. Electrodes were attached for polysomnographic monitoring to determine sleep stages. These included EEG, EMG, EOG, respiration by respiratpry belt and nasal thermistor, and oximetry. Starting at 1900, 5-ml blood samples were drawn every hour until 1900 on the next day. Thoughout this period subjects were either in a supine or a sitting position in individual rooms where they could read, use their personal computers or watch television. From 2300 to 0800 the room lights were turned off during the sleep period. Blood samples were collected into EDTA treated tubes, immediately centrifuged at 4°C, and plasma was stored at -70°C until assay. Hourly blood sampling during sleep continued with minimal disturbance to subjects' sleep. To standardize food consumption and to provide adequate energy intake, subjects received every 3 hours 150 ml of specially designed liquid food (Ensure^® ^formula) with the following composition: proteins (5.49 g, 84% caseinate, 16% soy – 14.7% of the calories), fat (5.3 g, 32% of calories), carbohydrates (20 g, 77% corn syrup, 23% sucrose, 53.3% of calories), vitamins and minerals, in 77 ml water. No other food except for water was allowed.

### Experiment 2

Thes second experiment was identical to Experiment 1 except for the fact that the sleep period was delayed from 2300-0800 to 0720-1500. As before, subjects were admitted to the laboratory at 1800 and blood was withdrawn every hour starting at 1900 until 1900 on the next day. Sleep was monitored polygraphically as described before. Food was provided as in Experiment 1.

### Experiments 3 and 4

In these experiments, we conducted a methionine loading test at two times: 0900 and 2100. The selection of these times was based on the results of the first two experiments that demonstrated a daily nadir and a nocturnal peak in Hcy levels (see below). At the start of each methionine loading test, subjects were administered 100 mg/kg body weight methionine, mixed in fruit juice. Blood samples (5 ml) were taken into EDTA treated tubes before methionine loading, designated as time 0, and then at +2, +4, +6 and +8 hours after methionine administration. Light carbohydrate rich meals were provided at +1 and +6 hours after the methionine loading in each of the test periods.

### Measurement of amino acids and vitamins

Plasma amino acids levels (Hcy, methionine, leucine, isoleucine and tyrosine) were measured in duplicates using a Biochrom 20 Amino-Acid analyzer (Pharmacia Biothech, Cambridge, UK) as described before [5]. The mean intra-assay CV was less than 3%. All samples from a single individual were analysed in a single run. In view of their involvment in Hcy metabolism, serum levels of folic acid and vitamin B_12_ were also measured in all samples of all subjects using commercially available kits from Abbott. The assays were performed on an Abbott IMX analyzer that utilizes ion capture technology for folate determination and microparticle enzyme immunoassay (MEIA) technology for B_12_. The assays were performed according to the manufacturers' instructions and used quality control sera supplied by Abbott.

### Statistical analysis

Repeated measurements ANOVA was used to compare the means of the amino acids between the first two experiments. To obtain the average 24-h Hcy curves, each individual data point was replaced by a z-transformation based on the individual 24-h mean and standard deviation, before averaging across subjects. Then, each of the individual time series was subjected to Cosinor analysis to determine its amplitude and acrophase. Since Experiment 1 was perfomed during the summer (August) and Experiment 2 was performed during early winter (late November), approximately 2 months after the change from Summer daylight-saving time to Winter time, during which the clock in Israel was advanced by one hour, the 24-h curves of the first experiment were advanced by 1 hour before the analysis. Then repeated measurements ANOVA was used to determine differences in acrophase between the experiments. In the third and fourth experimens, the concentrations of Hcy at times 0, 2, 4, 6, and 8 hours after methionine loading were analysed by repeated measures ANOVA to determine if there were any significant morning-evening differences in Hcy levels.

## Results

All subjects successfully completed the four experiments. In experiment 1 when they slept from 2300 to 0700, average sleep latency was 22.2 ± 7.3 min, total sleep time was 407.3 ± 51.8 min, and sleep efficiency was 77.7 ± 9.2%. In experiment 2 when they slept from 0720 to 1500, average sleep latency was 4 ± 3.1 min, total sleep time was 371.5 ± 59.4 min, and sleep efficiency was 83.6 ± 12.2%. In spite of the reversal of the sleep-wake cycle, the 24 h means and coefficients of variation of Hcy in the two experiments were very similar to each other, 8.82 μmol/L and 29.7% and 8.51 μmol/L and 27.7%, in experiments 1 and 2, respectively. None of the subjects had abnormal Hcy levels (>15 μmol/L) at any point across the 24 hours.

**Figure 1:**
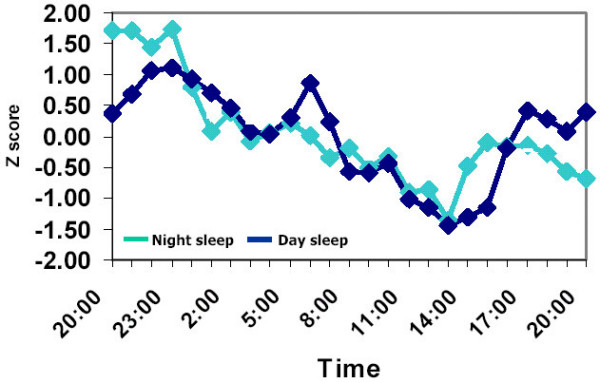
Daily rhythms in plasma concentration of Homocysteine. Rhythms were measured in 6 subjects who slept from 23:00 to 07:00 (Night sleep) or from 07:20 to 15:00 (Day sleep). Blood was withdrawn every hour starting at 19:00 until 19:00 the next day. Individual data points were transformed to Z-scores before averaging across subjects. For clarity purposes standard errors of data points are not presented. Magnitude of standard errors was approximatly 10% of mean values.

Figure 1 presents the average z-transformed 24-h curves of Hcy in the two experiments. In spite of the reversal of the sleep-wake cycle, the 24-h pattern of Hcy was remarkeably similar. In both experiments there was a midday nadir and a nocturnal peak in Hcy levels. In absolute terms, the daily rhythm in Hcy represents a change from nadir to peak values of 6.7 to 9.83 μmol/L (46.7%) and 7.4 to 10.55 μmol/L (42.6%), in experiments 1 and 2, respectively. Analysis of variance showed no significant difference in the average amplitude of the z-transformed rhythms of the two experiments, as determined by the cosinor analysis: 0.81 ± 0.19, and 1.07 ± 0.22 μmol/L, for experiment 1 and 2, respectively. There was, however, a significant difference between the timing of the average acrophase which was earlier by approximately 2 hours in experiment 1 than in experiment 2 (22:47 ± 0:45 vs. 0:54 ± 1:14, t = 3.77; p < .01).

**Figure 2:**
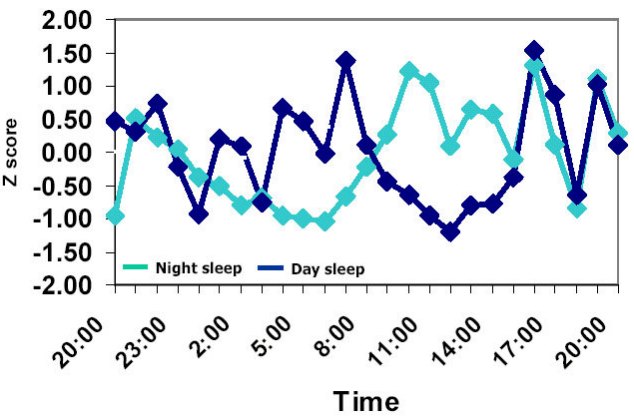
Daily rhythms in plasma concentration of methionine. Rhythms were measured in 6 subjects who slept from 23:00 to 07:00 (Night sleep) or from 0720 to 1500 (Day sleep). Blood was withdrawn every hour starting at 19:00 until 19:00 the next day. Individual data points were transformed to Z scores before averaging across subjects. For clarity purposes standard errors of data points are not presented. Magnitude of standard errors was approximatly 10% of mean values. Note the large pulses in methionine concentrations that appeared in synchrony with the times of feeding.

Strikingly different 24-h patterns were observed for the other amino acids: methionine, leucine, isoleucine and tyrosine. In all, the average z-transformed 24-h curves revealed a strong influence of both the sleep-wake cycle and the feeding schedule. Their level was notably lower during the sleep period, regardless of its timing, and increased every two hours in synchrony with the times of feeding. This pattern is exemplified in Figure 2 for methionine. Identical patterns were observed for leucine, isoleucine and tyrosine (data not shown).

We did not find any evidence for rhythmicity in the concentrations of B_12 _and folic acid. While folic acid showed a linear increase throughout the study period, the 24-h pattern of B_12 _was rather constant with slight elevation during the night time (data not shown).

### Methionine loading

**Figure 3:**
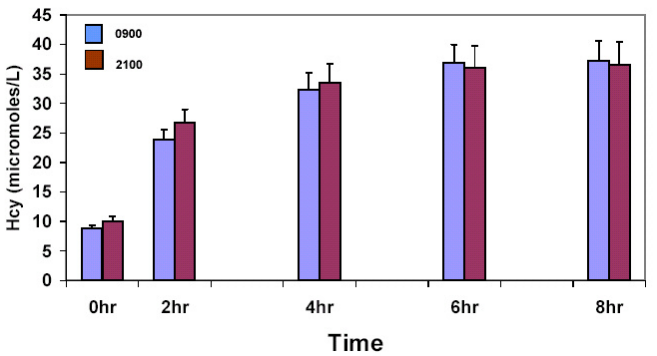
Plasma concentration of homocysteine before and after methionine loading. Shown are the means and standard deviations of plasma concentration of homocysteine in 6 subjects before (0 hr) and 2, 4, 6 and 8 hours after methionine loading at 09:00 and 21:00.

As expected, methionine loading resulted in increased plasma Hcy levels during both morning and evening experiments (Figure 3). Analysis of variance did not reveal overall significant differences between morning and evening post-methionine Hcy levels. However, inspection of Hcy levels at each of the time points separately revealed some interesting trends. Before methionine loading, as could be expected from the daily rhythm in Hcy found in experiments 1 and 2, morning Hcy level tended to be lower by 1.18 μmol/L than the evening level (p < .11, paired t-test, two tailed). Moreover, the increase in Hcy from time 0 to 2 hours after loading was greater by a mean of 2.8 μmol/L in the evening than in the morning (p < .09, paired t-test, two tailed). This resulted in evening and morning levels of Hcy of 26.66 and 23.86 μmol/L, respectively. These differences became much smaller at +4, +6 and +8 after the loading.

## Discussion

The present study demonstrated that under strictly controlled dietary conditions plasma levels of Hcy shows significant daily rhythmicity, which is independent of the 24-h cycle of sleep and wake, with a peak at around 2200 to 2400. Previously, similar rhythmicity in Hcy with an evening peak was reported in obese diabetic patients by Bremner et al [12] and with nocturnal peak in rats by Baydas et al [13]. We further extended these findings by demonstrating that daily rhythms exist also in normal young adults. In contrast to Hcy, there was no daily rhythmicity in methionine, leucine, isoleucine and tyrosine, in which the 24-h pattern followed both the timing of sleep and the feeding schedule.

Homocysteine is a non-protein sulfur containing amino acid, and an intermediate in the metabolism of the essential amino acid methionine. The metabolism of Hcy is accomplished by two major pathways, remethylation into methionine and transsulfuration to cystationine [14]. In remethylation, Hcy acquires a methyl group from N-5-methyltetrahydrofolate or from betaine to form methionine. The reaction with N-5-methyltetrahydrofolate is vitamin B_12 _dependent while the reaction with betaine is not. In the transsulforation pathway, Hcy condenses with serine to form cystationine in an irreversible reaction catalyzed by the pyridoxal-5'-phosphate (PLP)-containing enzyme, cystationine beta synthase. Although we do not have any information as yet on the underlying mechanism responsible for the daily rhythm in plasma Hcy, it is most probably related to the balance between its rates of production and disposal. A high Hcy concentration could be due to an elevated production rate, a decreased rate of transsulforation, a decreased rate of remethylation to methionine, or any combination of these processes.

The fact that the range of the daily variations in the plasma levels of Hcy is on the same order of magnitude as those seen in mild hyperhomocysteinemia, may suggest that the two phenomena share a common underlying mechanism. Mild hyperhomocystenemia seen under fasting conditions is due to mild impairement in the methylation pathway. This may be caused by folate or B_12 _deficiencies, or by methylenetetrahydrofolate reductase thermolability. The variations in plasma vitamin concentrations, however, could not provide an explanation for the daily rhythms in Hcy. The 24-pattern of folate levels showed a linear increase from the beginning to the end of the study. Although the plasma concentrations of vitamin B_12 _varied across the 24 hours – in contrast however to what was expected if B_12 _were involved in the daily rhythm in Hcy, ie, increasing levels of B_12 _associated with decreasing levels of Hcy – the 24-h pattern in B_12 _was parallel to that of Hcy with a daytime nadir and a night time peak. Thus, it is unlikely that a daily rhythm in plasma vitamin concentrations can  explain the daily rhythm in Hcy.

The methionine loading test has been used to test the individual's ability to dispose of methionine through the transsulforation pathway [14]. The fact that the differences between Hcy levels after morning and evening methionine loading were rather small and limited to the first 2 hours after the loading may indicate that the transsulforation pathway does not play a role in generating Hcy rhythmicity.

A different possibility that cannot be ruled out at this point is the involvement of the Hcy cellular export mechanism. The small amount of plasma Hcy is the result of a cellular export mechanism that is essential for keeping intracellular concentrations low to avoid potentially Hcy cytotoxic effects. Thus the daily rhythm in plasma Hcy may reflect variations in the activity of the cellular export mechanism, which result in varying levels of Hcy disposed to the plasma at different phases of the 24 hours rather than in its rate of metabolism. Further studies are needed to test this possibility.

Finally, what may be the clinical implications of the present findings? We would like to suggest that the existence of a daily rhythm in Hcy concentration may have possible health-related consequences to shift workers, who were shown to be at an increased cardiovascular risk [15]. Firstly, reversing the meals' schedule to a nocturnal orientation such that the time of major meal coincides with the time of the physiological peak of Hcy may have at least transient cardiovascular consequences. It was shown that an increase in Hcy concentration rapidly induces impaired elasticity of the coronary microvascular and central arterial circulation [16,17], conditions predictive of increased cardiovascular events rate [18]. Furthermore, even small physiological increments in Hcy concentration, induced by low-dose methionine or dietary animal protein meals that are more relevant to shift workers, induce a dose-related graded impairement in endothelial functioning [19]. Thus, consuming methionine or animal-protein-rich foods during the middle of the night may result in a greater risk of severe transient impairment in endothelial function than when a similar meal is consumed at the habitual lunch time during the day. Although we did not find significant differences in Hcy concentrations after methioning loading at 0900 and 2100, as expected, morning levels tended to be lower, and the initial increase in Hcy during the first 2 hours after loading was greater by a mean of 2.8 μmol/L in the evening than in the morning. This difference bordered on statistical significance. It is possible that, had we performed the methinine loading closer to the time of the nocturnal peak in Hcy, between 10 PM and midnight, this day-night difference would have been larger.

Secondly, we do not know how the desynchronization between the circadian system and the enviornment which occurs in rotating shift workers may affect the rhythm in Hcy concentrations and its overall plasma concentration. Recently, Martins et al [20] reported that long-haul bus drivers working shifts had higher concentrations of Hcy than a control group of day workers. In a study just completed in our laboratory we found that rotating shift workers who complained of disturbed sleep had significantly higher concentrations of Hcy than permanent day workers, or shift workers without sleep disturbances (paper submitted to press). Furthermore, life-style related factors like smoking and heavy coffee consumption that were shown to be associated with increased Hcy concentration [21,22], are more prevalent among shift workers than among day workers [23], and may also contribute to increased Hcy concentration. Of note, decreasing levels of melatonin induced by pinealectomy in rats were reported to be associated with increased plasma concentrations of Hcy, while treatment with exogenous melatonin restored it to basal concentrations [24]. Thus, suppression of melatonin by bright light during night work may be also associated with increased Hcy concentration.

In view of the fact that Hcy is a risk factor for cardiovascular morbidity, more research is needed on the possible role of hyperhomocysteinemia as a cardiovascular risk factor in shift workers.

## Conclusions

Our results demonstrated a daily rhythm in plasma concentrations of Hcy with a nocturnal peak that was independent of sleep-wake cycle and food consumption. There were no comparable rhythms in the concentrations of methionine, leucine, isoleucine and tyrosine, nor in the concentrations of B_12 _and folic acid. Methionine loading at 9 AM and 9 PM produced a comparable time-dependent increase in Hcy concentrations with a tendency toward a higher increase in the evening during the first 2 hours after loading. In view of the possible involvement of Hcy in cardiovascular morbidity, and of the increased cardiovascular morbidity in shift wokers, these findings may have implications to shift workers health.

## List of abbreviations

Hcy – homocysteine

EEG – Electroencephalography

EMG – electromyography

EOG – electrooculography

EDTA – ethylanediaminetetraacetic acid

CV – coefficient of variation

ANOVA – analysis of variance

## Competing interests

None declared.

## Author's contribution

PL and LL co-designed the study, supervised the data collection and data analysis and wrote the paper.
